# Cost-benefit parameters in evaluating automation

**DOI:** 10.1155/S1463924683000528

**Published:** 1983

**Authors:** Thomas M. Craig

**Affiliations:** Clinical & Instrument Systems Division E. I. du Pont de Nemours & Company Inc. Wilmington Delaware 19898 USA


Journal of Automatic Chemistry, Volume 5, Number 4 (October–December 1983), pages 210–211

### Cost-benefit parameters in evaluating automation

**Thomas M. Craig**

*Clinical & Instrument Systems Division, E. I. du Pont de Nemours & Company, Inc., Wilmington, Delaware 19898, USA*

### Introduction

In judging the value of automation, it is important to consider economic aspects and its impact on the laboratory, as well as on the hospital system.

Consequently, the objective of this paper is to present some useful financial parameters for evaluating the impact of automation in the clinical laboratory, plus some insight into the potential effect of automation on the hospital system.

### Key laboratory parameters

In order to evaluate the potential cost-savings of automation, it is necessary to first determine present costs in the laboratory. This provides the basis for comparing alternatives.

Total direct cost per reportable patient result is the best basis for comparison (total direct cost is defined as the reagents, reagent wastage, quality control [standard curves and daily quality control], glassware and other supplies, repeats and duplications, and labour required to perform a given group of clinical tests, divided by the volume of the tests being automated). When evaluating an automated alternative, one should also include in direct costs service contracts, instrument depreciation, as well as any costs required to modify the laboratory prior to accepting the automation. The cost of automation can be compared with present costs of performing the same group of tests given three levels of data, ranging from broad to specific, depending on the accounting or financial data available or obtainable. These include: (1) total laboratory costs divided by total reportable results; (2) total cost by laboratory area divided by total reportable results from that area; and (3) direct cost of all elements specific to the group of tests considered for automation. Obviously, the third level of data, which pinpoints direct costs specific to given laboratory procedures, provides the greatest accuracy level for comparison. For example, if automation is considered for the chemistry laboratory, all direct costs relating to the chemical tests to be automated should be determined.

Table 1 gives an idea of these costs by the major cost elements of reagents, quality control and labour in typical, small US hospitals' clinical chemistry laboratories. The cost figures are based on results of in-depth studies of clinical laboratories that primarily performed routine chemistry testing prior to automation.

*Table 1. Manual clinical chemistry testing cost per reportable patient result.*

| Hospital's annual chemistry test volume | No. 1 13M | No. 2 12M | No. 3 37M |
|---|---|---|---|
| Reagents, reagent wastage, and other supplies | \$0·45 | \$0·42 | \$0·56 |
| Quality control, repeats, and duplications | 0·63 | 0·89 | 0·72 |
| Labour | 2·11 | 1·55 | 1·59 |
| Total | \$3·19 | \$2·86 | \$2·87 |

Averaged together, these three clinical laboratories spent the least amount on reagents, reagent waste and other supplies (an area that most people focus the greatest attention on), a fourth on quality control, repeats and duplications, and the most on labour prior to automation (table 2).

*Table 2. Average percentage of clinical chemistry testing cost by cost element prior to automation.*

| | |
|---|---|
| Reagents, reagent wastage and other supplies | 16% |
| Quality control, repeats and duplications | 25% |
| Labour | 59% |
| Total cost | 100% |

Automation, in all three cases studied, reduced the total cost per reportable test (table 3). Automation costs included costs for instrument depreciation and service.

*Table 3. Total chemistry test cost/reportable result.*

| | Manual | Automation |
|---|---|---|
| Hospital No. 1 | \$3·19 | \$2·17 |
| Hospital No. 2 | \$2·86 | \$2·42 |
| Hospital No. 3 | \$2·87 | \$1·96 |

### *Impact on quality control*

Automation can improve the quality of clinical tests because it is typically about four times more precise than manual procedures. With greater precision, automated procedures were able to reduce quality control and the number of tests repeated and run in duplicates to a third of its former level at the six small hospitals where this was measured (figure 1).

### *Impact on labour productivity*

Automation can increase labour productivity by 93% to as much as 472%, according to results of this study of selected hospital clinical laboratories (table 4).

By increasing the productivity of the existing staff, automation reduces the need to add personnel when the laboratory's workload increases. Also, it improves workload distribution and allows the laboratory to use people with less training to process tests. This frees skilled technologists for more demanding tasks.

Space saving is another parameter to consider when evaluating automation. By reducing bench and chemical storage space, automation can reduce the size of the laboratory or offset the need to expand the laboratory with an increasing workload. This translates into major cost-savings because most laboratories operate on a cost-per-square-foot basis. In the US, for

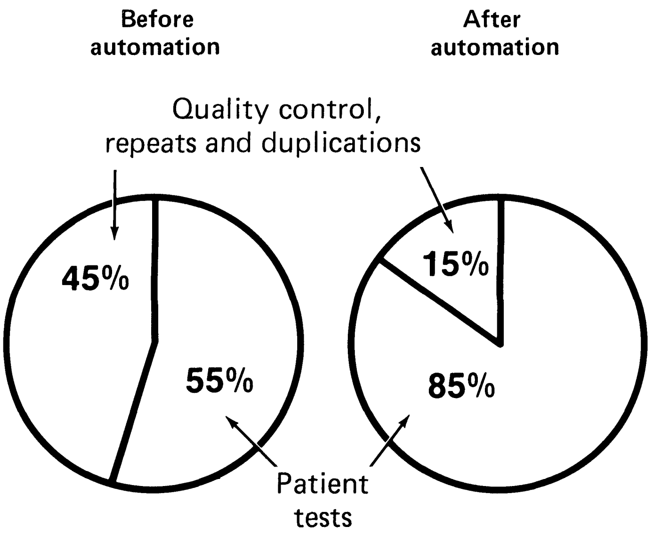

*Figure 1. Reduced quality control, repeats and duplications.*

*Table 4. Impact of automation on labour productivity.*

| | Chemistry testing Full time equivalent (FTE) | Chemistry volume | Productivity (tests/FTE) |
|---|---|---|---|
| Hospital No. 1 | | | |
| Before automation | 2 | 12 972 | 6486 |
| After automation | 2 | 25 934 | 12 967 |
| Hospital No. 2 | | | |
| Before automation | 1·5 | 12 236 | 8157 |
| After automation | 0·5 | 19 245 | 38 490 |
| Hospital No. 3 | | | |
| Before automation | 2·77 | 36 804 | 13 300 |
| After automation | 2·76 | 71 316 | 25 800 |

example, one study has shown that the average annual charge to a clinical laboratory is $280 per square foot. By increasing the number of tests performed per square foot, automation has had a dramatic impact on holding down laboratory overhead costs.

### Financial impact on the hospital system

Automation not only increases laboratory efficiency, but it also benefits the total hospital system. It speeds up laboratory turnaround time, which can increase patient turnover. It improves out-patient service by providing faster and more timely diagnostic information.

These benefits are most evident in small hospitals where it is easier to isolate the impact of automation on the hospital system. Studies in three small hospitals resulted in the following assessment of the impact of chemistry automation on the hospital system by the Hospital Administrators:

*Hospital Administrator No. 1*: 'reduction in the length of patient stay by two days and faster testing is certainly one of the reasons'.
*Hospital Administrator No. 2*: 'Having test results available faster has resulted in more people being treated as out-patients, shorter stays in the Intensive Care Unit (ICU), and quicker discharges... average patient stay is down 27%'.
*Hospital Administrator No. 3*: 'Our average patient stay has been reduced a full day since we got chemistry automation. Before adding chemistry automation, only about 6% of chemistry tests were for out-patients. Today almost one-quarter of all our chemistry tests are for out-patients. This is strong proof, we feel, in the effectiveness of quick-test technology to reduce the need for many people to become inpatients, often merely to await test results'.

The US government, in an effort to reduce the increase of the rapidly rising cost of inpatient care, has encouraged hospitals to take steps to reduce the length of patient stay and to treat patients on an out-patient versus inpatient basis. The three Hospital Administrators that we interviewed believe that chemistry automation has been a contributing factor to improving their institution's performance in both inpatient stay reductions and increasing the less expensive out-patient treatment.

### Conclusion/summary

Even in smaller clinical laboratories, automation can be cost-effective. In addition to reducing total direct cost per patient test by reducing quality control, and increasing labour productivity, there are indications that automation may, at least in part, assist the hospital system in reducing costs through shorter length of stay for inpatients and the ability to provide more services on an out-patient basis.

### READER ENQUIRY SERVICE

Each advertisement and each editorial item in the Product News section in *Journal of Automatic Chemistry* carries a number which corresponds with a number on the Reader Enquiry Card bound into every issue.

If there are any items on which you would like further information, circle the relevant number on the card, indicate the volume and issue number, fill in your name and full address, and return the card to Taylor & Francis Ltd. Your card will then be forwarded to the company/ies concerned for action.

*Through using the Reader Enquiry Service, you can obtain information on a variety of products through one simple operation.*

### EDITORIAL NOTE

The Editor is pleased to receive new books on chemical automation and mechanization. Review copies should be sent to:

*Dr. Peter B. Stockwell, P.S. Analytical Ltd, 2 Eagles Drive, Tatsfield, Westerham, Kent TN16 2PB, UK.*

